# Promoting insect farming and household consumption through agricultural training and nutrition education in Africa: A study protocol for a multisite cluster-randomized controlled trial

**DOI:** 10.1371/journal.pone.0288870

**Published:** 2023-07-19

**Authors:** Mohammed Hussen Alemu, Afton Halloran, Søren Bøye Olsen, Jacob Paarechuga Anankware, Philip Nyeko, Monica Ayieko, Evans Nyakeri, John Kinyuru, Silvenus Konyole, Saliou Niassy, James Peter Egonyu, Geoffrey Maxwell Malinga, Jeremiah Ng’ang’a, Charles Adino Ng’ong’a, Nicky Okeyo, Shadrack Kwaku Debrah, Samuel Kiiru, Amos Acur, Nanna Roos

**Affiliations:** 1 Department of Food and Resource Economics, University of Copenhagen, Copenhagen, Denmark; 2 Department of Nutrition, Exercise and Sports, University of Copenhagen, Copenhagen, Denmark; 3 Department of Horticulture and Crop Production, University of Energy and Natural Resources, Sunyani, Ghana; 4 Department of Forestry, Biodiversity and Tourism, Makerere University, Kampala, Uganda; 5 Department of Plants, Animals and Food Sciences, Jaramogi Oginga Odinga University of Science and Technology, Bondo, Kenya; 6 Department of Biological Sciences, Jaramogi Oginga Odinga University of Science and Technology, Bondo, Kenya; 7 Department of Food Science and Technology, Jomo Kenyatta University of Agriculture and Technology, Juja, Kenya; 8 Department of Nutritional Sciences, Masinde Muliro University of Science and Technology, Kakamega, Kenya; 9 International Center of Insect Physiology and Ecology, (*icipe*), Nairobi, Kenya; 10 Department of Biology, Faculty of Science, Gulu University, Gulu, Uganda; Szechenyi Istvan University: Szechenyi Istvan Egyetem, HUNGARY

## Abstract

**Background:**

Edible insects are a sustainable source of high-quality animal protein. Insect farming is gaining interest globally, particularly in low-income countries, where it may provide substantial nutritional and economic benefits. To enhance insect farming practices in Africa, new farming systems are being developed. However, knowledge on how to best promote uptake of these systems is lacking. This study aims to fill this gap by investigating the effectiveness of educational interventions in promoting insect farming for household consumption in Africa.

**Method:**

The study is designed as a multi-site randomized controlled trial to evaluate the impacts of agricultural training alone or in combination with nutrition education on the adoption of insect farming in Ghana, Kenya and Uganda. In each of the three countries, ninety-nine villages are randomly assigned to one of three arms: two intervention arms and a control arm with no interventions. Focusing on production (P), the first intervention arm covers agricultural training on insect farming combined with provision of insect production starter kits. Focusing on both production and consumption (PC), the second intervention arm involves the same intervention components as treatment P plus additional nutrition education. The impacts of the interventions are measured by comparing baseline and endline data collected one year apart. Primary outcomes are adoption of insect farming and consumption of the farmed insects.

**Discussion:**

Understanding the drivers and impacts of novel agricultural practices is crucial for transitioning to sustainable food systems. The current project is the first to investigate how educational interventions promote insect farming for household consumption in low-income countries. The results will contribute evidence-based knowledge to support sustainable development through insect farming in Africa.

**Trial registration:**

The protocol is registered in the American Economic Association registry for randomized control trials with registration number AEARCTR-0009996. Initial registration date: 02 September 2022, last updated 17 May 2023.

## Introduction

The vast majority of agricultural production in Sub-Saharan Africa is dependent on rain, making it highly vulnerable to the effects of climate change such as increasing frequency and severity of droughts [1]. This is expected to increase food insecurity and malnutrition [2]. At the same time, rapid population growth in Africa is driving an increasing demand for nutritious and healthy foods. Satisfying this demand in the face of climate change calls for a transition towards more sustainable and resilient agricultural practices [3], which is key to safeguarding livelihoods and improving food security and nutrition in the long term [4–7]. For smallholders, this transition involves finding and employing alternative low-cost farming approaches that have minimal land, water and feed requirements [3, 5, 6].

Farming edible insects is an alternative livestock production system with multiple potential benefits [5, 8, 9]. First, insect farming can assist nutrition improvement activities in food-insecure areas, as insects are generally rich in high-quality animal protein and essential micronutrients [10]. Second, compared to other livestock production, insect farming is characterized by lower greenhouse gas emissions. Additionally, it holds the potential to produce food that is nutritionally equivalent to livestock while requiring less water, land, and other inputs. These potentials make insect farming resilient to the anticipated effects of climate change [11, 12], positioning it as a climate-smart agricultural practice [13]. Third, insect farming may support rural livelihoods in Africa by creating employment opportunities and generating cash income for households [14]. Despite these potential benefits, insects have mainly been harvested in the wild and consumed seasonally or sporadically in traditional diets in many parts of the world [15]. This practice of wild harvesting has led to overexploitation of insect specifies, and the consumption of insects in traditional diets is declining globally, largely due to the influence of westernized eating habits [16–18]. To address these challenges, insect farming has emerged as a viable solution to promote insect consumption through sustainable production systems [5, 16]. Furthermore, the growing emphasis within the agricultural sector to meet the increasing demand for nutritious and environmentally sustainable foods has highlighted the potential of insect farming as a novel approach to animal-based food production globally [4, 5, 8].

Compared to other agricultural production sectors, insect farming is relatively young and most producers operate on a limited scale [14, 19]. Legislative barriers often impede growth of this sector [20]. However, some progress has been made in improving legislative frameworks, especially in Africa [9]. For instance, in Kenya and Uganda, insects are recognized as indigenous foods, and standards have been developed for their regulation [21, 22]. The development and promotion of insect farming has so far had limited anchorage in research and innovation [9]. However, stakeholders in private and public sectors are currently focusing on innovation and knowledge transfer activities to develop farming systems including rearing facilities and management practices suitable to various insect species [9, 23, 24]. This has led to the emergence of several new insect farming systems around the world [5, 9, 14]. For instance, cricket farming has been developed and successfully implemented in Thailand [24, 25]. Similar farming systems are currently being adopted and promoted in Africa to produce insects on small, medium and large scales [5, 9, 26]. A recent country-level rapid survey conducted by the World Bank reveals that insect farming is practiced in 10 out of the 13 surveyed countries in Africa [5].

Several studies have explored various aspects of utilizing insects as human food, and their findings have been compiled in review studies. These studies suggest that consumers in developing countries generally exhibit positive attitudes towards insects as a food source. Additionally, the studies emphasize that insects are a valuable and safe source of essential nutrients, which can be produced with minimal negative environmental impacts [10, 18, 27]. However, little is known about the most effective approaches to promote insect farming, especially in an African context. If the aim is to promote insect farming to support livelihoods and food provision in rural Africa, targeted interventions need to be identified and tested for their effectiveness. The current study conducts a randomized control trial (RCT) in three Sub-Saharan Africa (SSA) countries, namely Ghana, Kenya and Uganda, to investigate the effectiveness of educational interventions in promoting small-scale insect farming and household consumption. The focus on educational interventions was informed by previous studies indicating that lack of knowledge and skills in relation to both insect farming practices and nutritional benefits of consuming insects are the main causes of low uptake of insect farming for household consumption [21, 28]. By providing new, evidence-based knowledge on the promotion of small-scale insect farming and consumption in SSA, the study contributes to the advancement of appropriate policies for realizing the full potential of insect farming for sustainable development. Specifically, the study addresses several of the UN’s Sustainable Development Goals, including improving livelihoods (SDG1), improving nutrition and health (SDG2), supporting gender equality (SDG5), creating job opportunities (SDG8), reducing environmental impacts (SDG13), and promoting partnerships to achieve the different goals (SDG17) [5, 7].

## Methods and materials

### Study setting

The selection of Ghana, Kenya and Uganda for our study is justified as follows. First, consumption of insects collected from the wild is a part of local food cultures in all the three countries, providing nutritional and economic benefits to the population [29]. People in these countries are thus not completely new to the notion of eating insects. In addition, insect farming activities are emerging in the three countries driving innovation and sustainable development [5, 9]. Second, the multisite intervention study across an east-west gradient enables coverage of a variety of cultural contexts and agro-climatic conditions in SSA, enhancing the external validity and generalizability of our findings to other parts of SSA. Third, findings generated from this study can provide inputs for stakeholders involved in developing legislative and regulatory frameworks for the use of insect as food in the three countries. Thus, the multisite approach can strengthen the institutional frameworks needed to establish sustainable edible insect value chains and food systems in Africa. Fourth, our study involves three different insect species across the three countries, selected based on their likelihood of acceptability within the local culture and the availability of insect farming systems tailored to their specific life cycle and needs [23]. These species include Palm weevil larvae (*Rhynchophorus phoenicis*) in Ghana, Crickets (*Scapsipedus icipe*, *Acheta domesticus)* in Kenya, and African edible bush crickets (*Ruspolia differens Serville*) in Uganda [26, 30, 31]. The inclusion of diverse farming systems allows us to comprehensively analyze approaches to promote the adoption of farming practices and consumption of culturally appropriate insect species on a broader scale. The study thus contributes to the development of a generic framework for describing impact pathways of small-scale insect farming in relation to improving nutrition, health and livelihoods.

### Study design

The study involves a 1:1 cluster-RCT with two intervention arms. The first intervention arm focuses on insect production. It includes agricultural training on insect farming and the provision of insect production starter kits *(Treatment P)*. The second intervention arm is focused on insect production plus insect-based food consumption. It has the same intervention components as *Treatment P* plus an additional component focused on nutrition education and insect cooking demonstrations *(Treatment PC)*. A control arm with no interventions *(Control)* serves as a comparison group for the treatments. The effectiveness of the interventions is evaluated by comparing baseline and endline data collected one year apart.

### Program theory

**Fig 1:**
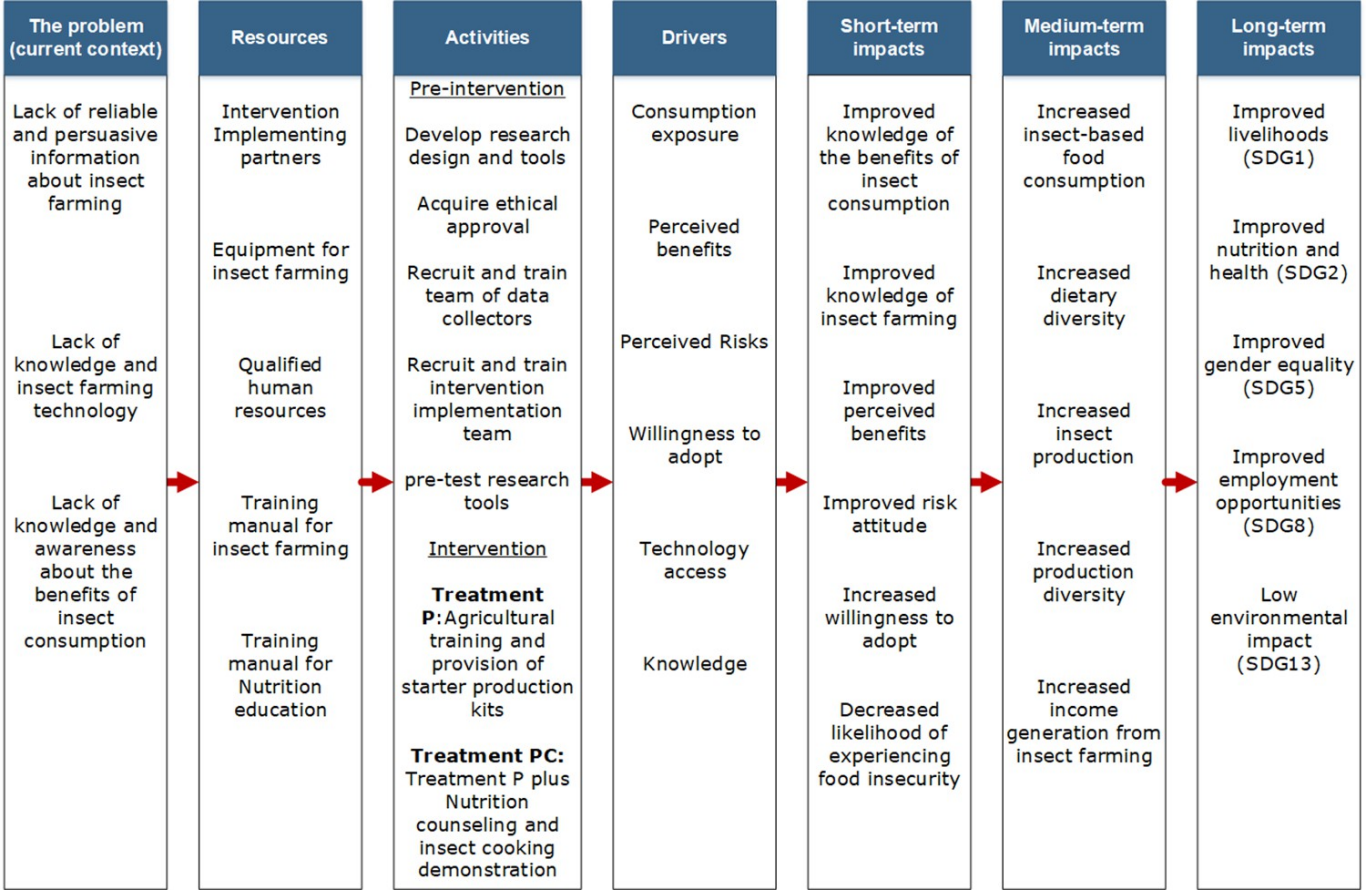
Conceptual framework showing pathways between the interventions and their outcomes.

Based on the literature identifying the complex pathways linking agricultural interventions and health and nutrition outcomes [32, 33], we develop a conceptual framework for the study as shown in Fig 1. The conceptual framework presents the problems, the resources and activities to tackle the problems, and the resulting impacts. The agricultural training components are expected to address the lack of knowledge about insect farming in rural communities and affects the drivers associated with knowledge, perceived benefits and risks, technology access, and willingness to adopt insect farming. The nutrition education and cooking demonstration interventions are expected to address the lack of knowledge and awareness about the benefits of insect consumption and the lack of insect-based food preparation skills. The interventions affects all the drivers in Fig 1 except for technology access. The short-term impacts of the intervention activities are likely to be improved knowledge of insect farming and cooking insect-based foods, and the benefits of insect consumption, positive perception of insect farming and consumption, decreased likelihood of experiencing food insecurity, and increased willingness to adopt. These short-term impacts can lead to medium-term impacts consisting of increased insect consumption, increased dietary diversity, insect production, and income generation. The combined short- and medium-term impacts can result in long-term impacts, which are part of the UN Sustainable Development Goals.

### Study aims and objectives

The overall aim of the project is to generate evidence regarding the effectiveness of educational interventions in promoting insect farming for household consumption in Africa. More specifically, the main objectives are:

1. To evaluate the impacts of insect farming and nutrition education interventions on household adoption of insect farming and consumption of farmed insects.
2. To develop an evidence-based framework describing impact pathways from promoting insect farming and consumption to outcomes related to insect production and consumption, with a particular focus on potential long-term development outcomes related to nutrition, health, and livelihoods.

### Sample size

To calculate the sample size, we determine the minimum detectable effect size based on the best available data in the literature regarding the effects of educational programs on adoption of insect farming. In Ghana, Kenya and Uganda, insect farming remains relatively uncommon compared to other types of farming activities. For instance, in a pilot study on cricket (*Scapsipedus icipe*, *Acheta domesticus)* farming in Kenya, only 12% of the sampled farmers adopted cricket farming [28]. We use this information to determine the minimum detectable effect size in our power analysis. We assume a minimum detectable effect size of 0.25 for Kenya, as an effect size of 0.12 would be too small to be detected given the available budget for the study. In Ghana, 560 farmers were trained as part of a pilot project focusing on farming Palm Weevil (*Rhynchophorus phoenicis*) production near their homes or gardens [34]. The same study reported that 48% of the trained farmers were actively farming Palm Weevil after the training. To avoid overestimation of the effect size, we consider a minimum detectable effect size of 0.25 for Ghana. In Uganda, pilot studies indicating the proportion of farmers involved in the farming of African bush crickets (*Ruspolia differens Serville*) were not found. Thus, we refer to related studies on the adoption of climate-smart agriculture practices in Uganda. Accordingly, we assume a minimum detectable effect size of 0.25 based on a previous study concerning climate-smart agricultural interventions [35].

**Fig 2:**
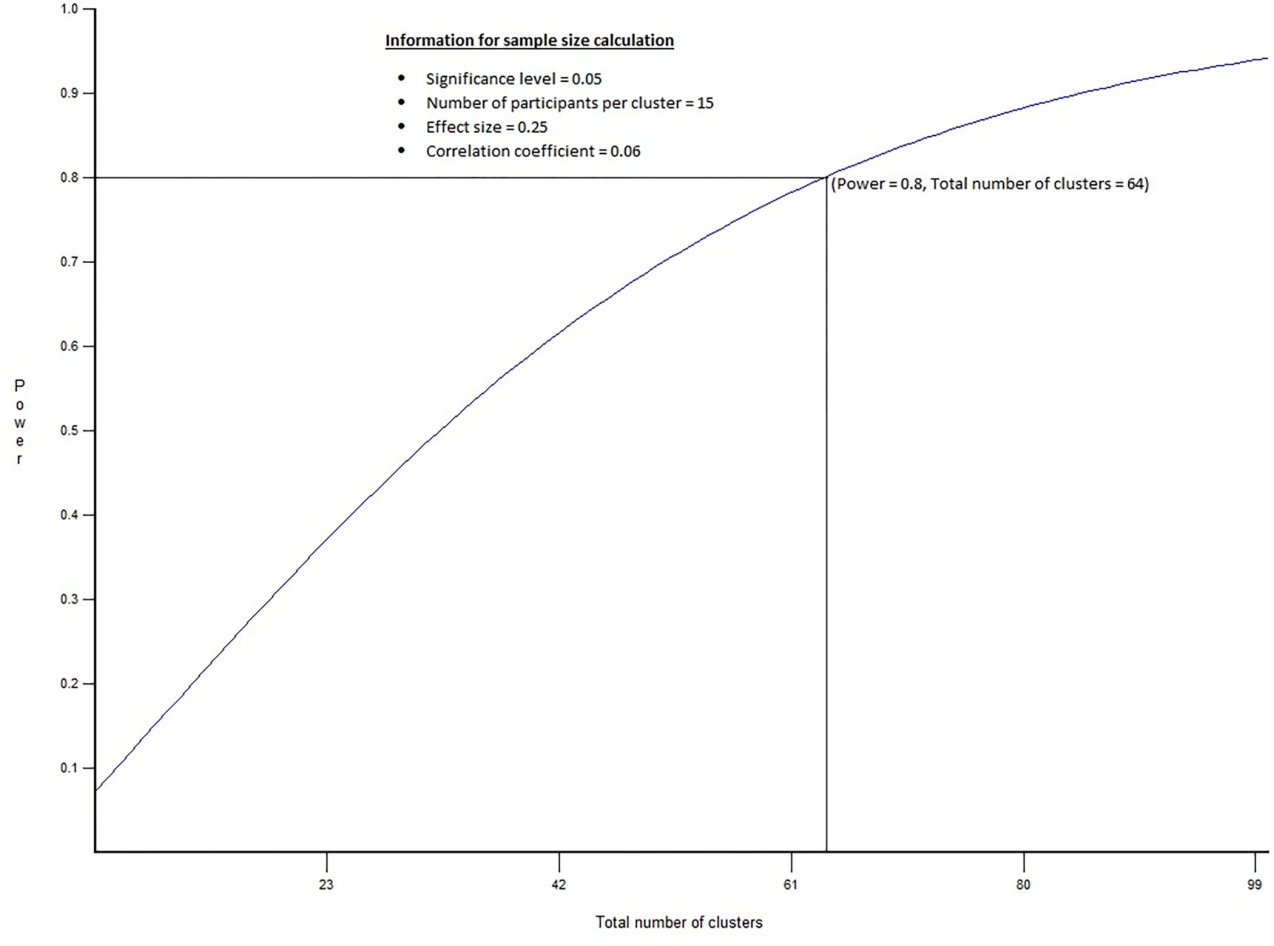
Power versus number of clusters.

Using the Optimal Design Software for multilevel and longitudinal research [36], the sample size is calculated based on a 0.8 power, 0.05 significance level and 0.06 intra-cluster correlation coefficient. Fig 2 shows that a total of 64 clusters (villages), each with 15 participants, are needed to obtain 80% of power for cluster randomized designs with two intervention groups. The minimum sample size across each of the intervention and control arms thus includes 32 villages in each country. To ensure that we detect the same effect size or smaller, we increase the sample size to 33 villages for each of the intervention and control arms.

Based on our experience from previous farming and nutrition intervention studies in the three countries, we expect an attrition rate of no more than 10%. Thus, the number of participants is increased by 10% to accommodate for attrition in our sample size calculation. To reduce the attrition rate, the implementation team visits the participants frequently to address any concerns related to the intervention. The implementation team also arrange alternative meeting schedules in case the participant is unavailable during endline surveys. Any missing values resulting from attrition are dropped from data analysis.

### Recruitment and randomization

**Fig 3:**
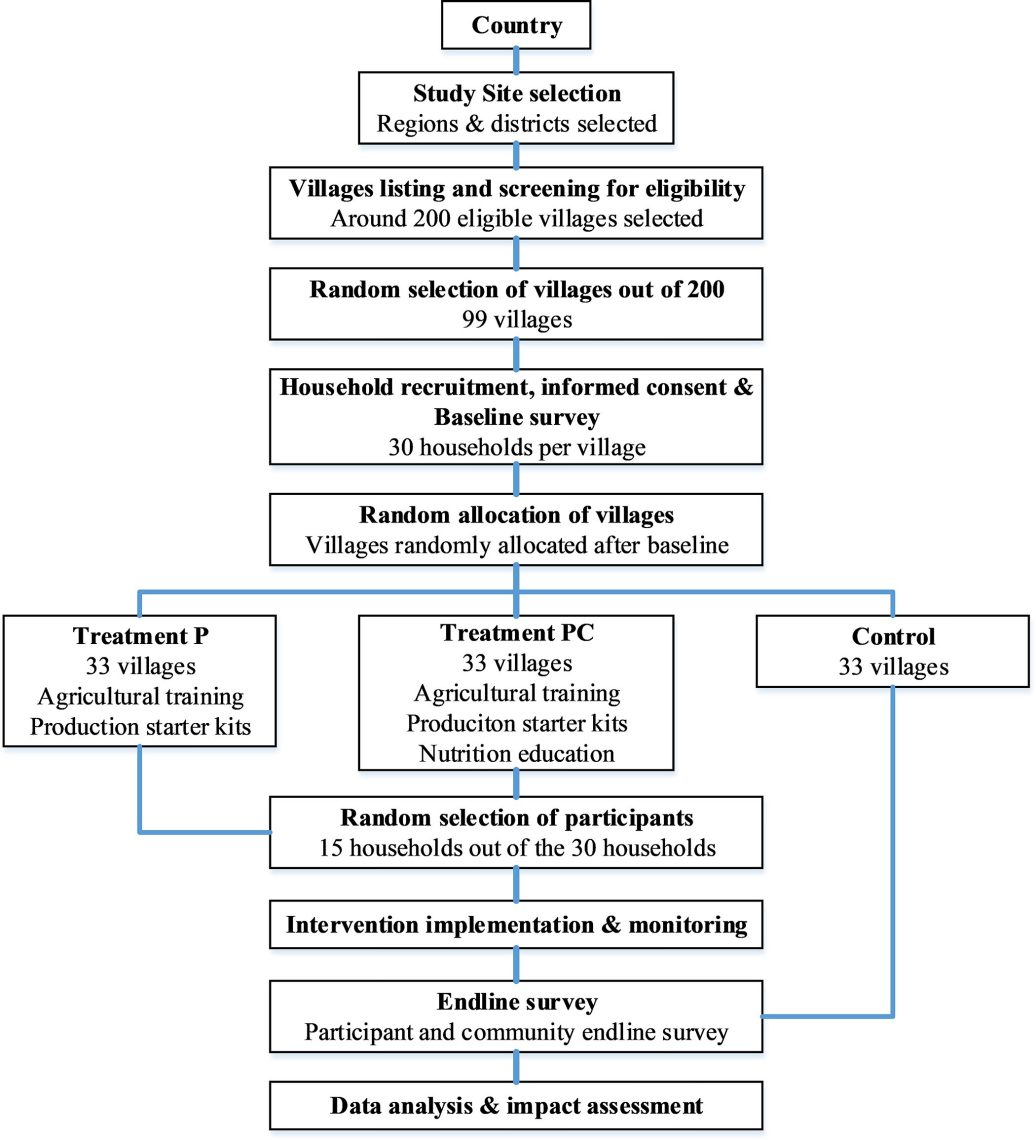
Flow diagram for the study.

A multilevel sampling procedure is used to identify and select the villages and participants included in the study (Fig 3). In terms of regions and districts, study sites are selected considering infrastructure accessibility and availability of rural areas with a large number of villages. In addition, areas with traditional insect consumption are selected to facilitate the adoption of insect farming. Previous studies on the adoption of new agricultural technologies and new food varieties suggested that previous consumption is an essential driver of adoption [25, 28, 37, 38]. Once the study sites are identified in each country, eligible villages are selected based on a range of inclusion and exclusion criteria. A village is eligible for the intervention if:

1. It has not been exposed to previous insect farming activities, including formal insect farming training
2. It has at least 60 households
3. It is not located adjacent to another village previously exposed to insect farming
4. There are no active agricultural intervention in the village

In addition, selected villages should not share common boundaries to reduce the risk of unintended spillover effects between treatment and control groups. Based on the above criteria, a list of 200 eligible villages is generated in Excel per country. Using Excel’s function to generate random numbers, 99 villages are randomly selected for the study in each country.

Households are selected and recruited based on a random walk door-to-door recruitment procedure as it is impossible to find a pre-existing list of households. Depending on the number of households in a village, every second or third household is approached for recruitment until a total number of 30 households in a village have been recruited to participate in a baseline survey. To be eligible, residents of the household must:

1. Have children below five years of age, as nutrition interventions usually target these groups of the population in Africa;
2. Live permanently at the same address;
3. Be able to participate in the agricultural training and nutrition education sessions;
4. Be able to perform insect farming and be able to provide the required space and housing for insect farming; and
5. Seek information to solve problems with his/her own agricultural production

The fourth criterion is needed because household insect farming is typically carried out indoors, either in a separate room or in a separate building close to the main house, to prevent theft of materials and protect against predators [21, 26]. Additionally, the availability of suitable space was identified to be one of the barriers to insect farming [28]. We assess farmers’ ability to provide space by observing the type of construction material used in their houses, specifically by determining whether the material consists of corrugated metal roofs and semi-permanent building materials. The fifth criterion is needed to acknowledge the fact that access to information plays a crucial role in driving the adoption of new agricultural technology by enabling farmers to understand potential risks and challenges, and take necessary precautions to minimize risk [39, 40]. Since insect farming represents a new farming system that may be perceived as a risky venture by farmers [28], which, in turn, could deter adoption, we include only households that actively seek information to solve problems with their own agricultural production. We measure farmers’ information-seeking behavior by asking them whether they seek information from sources such as agricultural extension offices, community groups (such as women’s groups and farmer group), churches/mosques, mass media, and the internet, when they face problems.

Once households are determined to be eligible, household heads are approached, and informed consent is sought. After obtaining consent, they are invited to participate in the baseline survey. Once the baseline surveys are completed and the relevant information is gathered, the 99 villages are randomly assigned to the intervention and control groups (see Fig 3) using computer-generated random numbers. Out of the 30 household heads participating in the baseline survey in each village in the two intervention arms, a simple random draw lottery is used to select 15 household heads to participate in the intervention. The remaining 15 household heads that are not selected for intervention still participate in the community endline survey (see Table 1). The purpose of this survey is to assess whether and how the information regarding insect farming and consumption has spread beyond the households included in the intervention [37].

**Table 1:** Number of clusters and participants per country.

Study arm	Clusters	Baseline survey	Intervention with participant endline survey			Community endline survey
	Villages	Participants^a^ per village	Participants per village	Participants per arm	Participants per arm (extra 10% added)^b^	Participants per village^c^
Treatment *P*	33	30	15	495	545	15
Treatment *PC*	33	30	15	495	545	15
Control	33	30	0	0	0	30

Note: ^a^ Participants are household represented by the head of the household.

^b^ The number of participants per treatment arm was increased by 10% to account for attrition.

^c^ The remaining 15 participants per village from the baseline survey who are not part of the intervention are included in the community endline survey. No interventions are implemented in the control villages, therefore, all the 30 household are included in this survey.

To ensure research integrity and methodological rigor, the village randomization process is blinded to all individuals involved in the research and households residing in the 99 villages prior to the intervention. A randomized sequence is generated in STATA [41] by a colleague not involved in the research. The outcome of the randomization is only revealed to project staff and households after the randomization process is completed. During the intervention, blinding is not maintained, except in the control villages where households are unaware of the intervention. Project staff members only visit control villages during the baseline and endline surveys, and no visits are conducted during the intervention to prevent information leakage and reduce potential biases that could arise from knowledge of the intervention in the control group.

### Intervention

To achieve the overall objective of the study, specific interventions focusing on agricultural training and nutrition education is implemented in *Treatment P* and *Treatment PC* for a period of 6 months. The components of these treatments are described in detail below.

#### Components of *Treatment P*

*Agricultural training on insect farming*. The agricultural training has been designed to teach intervention participants about small-scale insect farming. A training manual has been developed to ensure that the same information is transmitted to all participants. The training is based on behavior change techniques and key messages in relation to successful insect farming [42]. It covers a range of topics relevant to promoting insect farming for consumption. The topics are divided into six main elements, as shown in Table 2. Extensive training is provided in all villages where the *Treatment P* is implemented. The 15 participating household heads per village are divided into two groups during training sessions to ensure effective information dissemination to each participant and smooth feedback discussions. The training is conducted within one day and is organized into several sessions, scheduled to follow the main elements outlined in Table 2.

**Table 2:** Main elements of the agricultural training intervention.

Overall topic	Specific information
**1. Introduction**	• Welcome • Elements of the training sessions • Time schedule
**2. Overview of insects as nutritious and healthy food**	• Insects as food for human consumption • Benefits of insect farming
**3. Ecology and Biology of insects**	• Insect life cycle • Sex determination in insects • Insect behavior
**4. Starting an insect farm**	• Rearing house and site selection • Rearing equipment • Rearing container • Cage arrangement
**5. Management of an insect farm**	• Insect feeding and watering • Knowing signs and timing of egg laying • Preparing egg-laying substrate • Egg collection, incubation and hatching • Caring for pinhead • Hygiene maintenance • Pest and disease control
**6. Harvesting and post-harvest handling**	• Harvesting methods • Storage and transportation of insects • Post-harvest insect processing methods

The training activities begin with inviting the selected household heads to an information meeting. Here, participants are briefed on the purpose and nature of the intervention, what they can expect during the intervention, and what they are expected to do as participants. At this point, participants are made formally part of the intervention and assigned an identification number. Implementation teams are formed and receive training before commencing the interventions. They are responsible for providing the agricultural training according to the training manual. An implementation team consists of two persons assigned to half of the villages in each treatment arm and conducts the training on a rotating basis to avoid bias induced by the implementation team.

For example, if *Implementation Team 1* covers the first half of the villages in *Treatment P*, *Implementation Team 2* covers the first half in *Treatment PC*. Then, the team rotates, where *Implementation Team 1* covers the other half of the villages in *Treatment PC* while *Implementation Team 2* covers the other half in *Treatment P*. The training is given in a rolling sequence, i.e., the implementation team conducts training in one village at a time and continues until all villages are covered. The same implementation team visits each participant at least once a month to reinforce training messages and provide technical support for insect farming activities. These visits are important for participants as they give opportunities to raise questions and for the implementation team to tailor messages according to the specific contexts of the participant [43].

*Distribution of production starter kits for insect farming*. After the agricultural training, each participating household head receives a starter kit for free for small-scale insect farming. While the specific composition differs across the three countries due to differences in the types of insects selected for farming, the starter kits generally consist of live insects or eggs, insect rearing equipment and container, feed for insects, and a cleaning kit (see Table 3).

**Table 3:** Starter kits by country and insect species.

Country	Type of insect to be farmed	Main components of starter kits
**Ghana**	Palm weevil larvae (*Rhynchophorus phoenicis*)	• Adult larvae • Rearing container • Rearing equipment including rubber band, round bins, scale, stacker, shelves, aluminium mesh and rope • Start-up feeding substrate and a cleaning kit
**Kenya**	Crickets (*Scapsipedus icipe*, *Acheta domesticus)*	• A batch of eggs for hatching • Buckets • Cricket rearing equipment including cotton wool, spray bottle, egg hideouts, oviposition saucers, thermometer, and ant repellant • Starter feed and water dispenser, and a cleaning kit
**Uganda**	African edible bush crickets (*Ruspolia differens Serville*)	• Parent stock • Rearing cages • Rearing equipment, including egg-laying medium and egg hatching containers • Feed and water dispenser, and a cleaning kit

Each starter kit is expected to support 2–5 insect production cycles per participant with each cycle yielding 3–5kg of insects. The estimated cost of the starter kits is between 50–60 USD, 100–120 USD, and 10–15 USD per starter kit in Kenya, Uganda and Ghana, respectively.

The starter kits are distributed to participants on a rolling basis in each village immediately after the agricultural training and nutrition education sessions are completed. The kits are delivered directly to each household to ensure the participants receive them on time and get individualized advice on how and where to set up insect production on their land. The type, quantity and quality of starter kits are harmonized across participants to avoid a situation where the nature of the starter kits influences the impact of the intervention.

#### Components of *Treatment PC*

*Additional nutrition education component*. In addition to the intervention components in *Treatment P*, the *Treatment PC* includes the nutrition education designed to provide participants with specific nutrition knowledge about insects as food. This is expected to address the lack of information about the benefits of consuming insects, which in turn is expected to increase demand. Research suggests that demand creation is required for achieving high adoption rates of new food varieties [e.g. 37]. Nutrition education goes beyond only explaining the nutritional benefits of insects. It also addresses the lack of insect food preparation skills by providing demonstrations of how to prepare insect-based food. Specifically, households participate in live sessions involving cooking demonstrations and consumption of insect-based foods along with the provision of nutritional information. A manual for the nutrition education sessions is developed so that all participants receive identical information. The manual covers the topics shown in Table 4. Insects are expected to be consumed as whole cooked insects or they can be processed and blended with other conventional foods [44]. Thus, the cooking and consumption demonstration is based on porridge blended with insect flour, and whole cooked insects. The nutrition education component is implemented in villages assigned to *Treatment PC*. It is conducted within one day, comprising several sessions that cover the topics in Table 4. Households participating in the agricultural training are invited to attend the nutrition education training. As our focus is on households with children below five years of age, primary caregivers are specifically invited to participate, especially in cases where the head of the household is not the primary caregiver of the children. Similar to the agricultural training, implementation teams are formed and receive training to provide the nutrition education on a rotating basis. The implementation teams also provide refresher education when visiting participants in their households whenever necessary.

**Table 4:** Main elements of the nutrition education intervention.

Overall topic	Specific information
**1. Introduction session**	• Welcome • Time schedule • Elements of the training sessions • Aim and objective of training • Nutrition as a right • Importance of good nutrition to individuals, household and community
**2. Causes and consequences of nutritional deficiencies**	• Causes of poor nutrition • Consequence of poor nutrition • Effect of malnutrition to individuals • Preventive measures of malnutrition • Food groups and nutrition • Function of each food group • Breastfeeding • Contribution of animal source food to diet • How much to it (My plate)
**3. Nutritional benefits of insects**	• Nutritional benefits of insect consumption • Nutritional content of insects • Nutritional benefits of insects for child development • Food safety issues related to insects as human foods
**4. Insect cooking demonstration and consumption**	• Food processing • Insect food recipes • Cooking steps • Food tasting

#### Intervention monitoring plan

In line with previous protocols for RCTs [e.g. 43], we follow an intervention monitoring plan to record intervention activities and monitor outputs.

*Intervention activity records*. The intervention activity record involves tracking intervention activities. These include a) attendance of each participating household at agricultural training and nutrition education sessions; b) when and what topics were included in the sessions; c) length of each session; d) when production starter kits were delivered to each participant; and e) when implementation staff visits each participating household.

*Output monitoring*. Output monitoring involves a routine assessment of intervention outcomes during implementation [43]. The implementation team visits households participating in the interventions and conduct monitoring interviews and dialogue in each village.

### Outcomes

#### Overview of outcome variables and measurements

*Primary outcomes variables*. The primary outcome variables indicating the impacts of the interventions are: 1) whether farmed insects are consumed in the household, and 2) adoption of insect farming. The first primary outcome variable is measured during the baseline and endline surveys using questionnaires that inquire whether the participants cook and consume farmed insects. For participants, who cook and consume the farmed insects, they are asked to indicate the frequency of consumption within the last one year using a five-point scale ranging from 1–3 times per month to 1 or more times per day. The second outcome variable is measured through direct observation to determine whether the participant is still engaged in insect farming at the time the endline survey is conducted. Engaging in insect farming activities entails management of an insect farm, as well as harvesting and postharvest practices, as described in Table 2.

*Secondary outcome variables*. The secondary outcome variables are: 1) Insect farming and nutrition knowledge: is measured based on simple exam-like questions relating to the content of the agricultural training and nutrition education. In the questions addressing the agricultural training, participants are asked about their knowledge regarding starting an insect farm, managing an insect farm, and harvesting insects, as well as postharvest handling practices. In the questions addressing the nutrition education, participants are asked about their awareness of the nutritional content of insects and their importance for human consumption, as well as the insect-based food products that can be prepared and consumed by humans. This way of measuring knowledge enables us to effectively capture respondents’ ability to retain knowledge and memorize specific aspects of the agricultural training and nutrition education as a result of efforts made during and after the training. Our measurement of knowledge aligns with the suggestion by Kondylis, Mueller [45], and the existing literature on the adoption of climate-smart agricultural technologies in Africa [e.g. 37, 46, 47]; 2) Dietary diversity: is measured through child dietary diversity and household dietary diversity scores [48, 49]. Both scores are based on six food groups, including meat products, eggs, vegetables and fruits, starchy foods, dairy products, and pulses, legumes and nuts. Participants are asked to report whether and how often their children have consumed foods from each food group in the past seven days for the child dietary diversity score, and in the past thirty days for the household dietary diversity score; 3) Food insecurity perception: is measured based on the food insecurity experience scale presented in Cafiero, Viviani [50]; and 4) Perceived benefits and attitudes: is measured based on different questions focusing on peoples’ willingness to take risks in general, their risk attitude towards insects farming, and their willingness to adopt insect farming as an agricultural practice. The questions are developed based on relevant literature [e.g. 51–53]. Willingness to take risks is specifically measured using a ten-point scale from 0 (unwilling to take risks) to 10 (fully prepared to take risks) [51]. The risk attitude towards insect farming and willingness to adopt insect farming are measured using a five-point scale, ranging from strongly disagree to strongly agree. Information on all the aforementioned secondary outcomes are collected during both baseline and endline surveys.

### Data collection

Data is collected through two rounds of household surveys in each village on a rolling basis. As shown in Fig 3, the baseline survey is conducted in each village before villages are randomly assigned to the intervention and the control groups. Second, the endline survey is conducted after the end of the interventions to measure intervention impacts, which are measured using selected outcome variables. The endline survey has two components: participant endline survey and community endline survey (Fig 3). The former is conducted with the 15 participants (household heads) who take part in the interventions (Treatment P or Treatment PC) in each village. The latter is conducted with the remaining 15 household heads who participated in the baseline surveys but were not selected to participate in the interventions. Since no intervention is implemented in the control arm, all the 30 household heads who participated in the baseline surveys are included in the community endline survey for the villages allocated to this arm. Training is given to field data collectors (*survey team*) responsible for baseline and endline surveys. They are trained on the content of questionnaires, question-asking techniques and cultural sensitivity [43]. Supervisors are responsible for quality control in the field and addressing issues arising during data collection. Data collectors involved in baseline surveys are blinded in such a way that they are instructed not to share any information in the villages regarding the randomization of the villages into the intervention or control group. This is important to ensure that their interactions and communication with the villages do not unintentionally disclose any information about the intervention that could influence its later implementation.

#### Data source and data collection method

The data sources and collection methods are summarized in Table 4, developed and adapted based on Wendt, Sparling [43]. A questionnaire is developed to collect data during baseline and endline surveys. The baseline and endline surveys are made similar across countries but modified slightly to reflect differences between the local contexts. The endline survey follows the same structure as the baseline survey, except that it will be redesigned to ensure that the participants’ experiences regarding insect farming and consumption during the intervention are fully captured. The questionnaires have several sections, including socioeconomic and demographic status, child and household dietary diversity, insect farming and consumption knowledge, food insecurity perception, perceived benefits and risk attitude, and willingness to adopt insect farming and consumption. In addition to the baseline and endline surveys, data will be collected through intervention output monitoring while the intervention is being conducted (see Table 5).

**Table 5:** Data source and data collection method.

Activity	Data source	Data collection device	Responsible team	Population	Frequency
**Survey**	Baseline survey	Smartphone	Survey team	Treatment and control groups	Once per village
**Survey**	Endline survey	Smartphone	Survey team	Treatment and control groups	Once per village
**Monitoring**	Intervention activity records	Smartphone	Implementation team	Treatment groups only	After every intervention activity
**Monitoring**	Output monitoring	Smartphone	Implementation team	Treatment groups only	Bi-monthly in each village on a rolling basis

Note: table adapted from Wendt, Sparling [43]

#### Data management

Redcap (https://www.project-redcap.org/) is used to manage all data in the project. Data collection instruments are created using the same software, which allows detection and prevention of common data collection errors. It also enables storage and compilation of data as it comes in from the field. The research team and field supervisors are responsible for regular data quality checks. Any events of data concerns are communicated with the survey team and field supervisors for resolution. Access to data is restricted to principal investigators and the server where the data are stored is password protected to ensure confidentiality.

### Analysis plan

We focus on the intent-to-treat (ITT) effects. Some participants may not attend to the agricultural training or nutrition education sessions despite being invited to the sessions. In the ITT analysis, we estimate the effects of the interventions by including all participants irrespective of whether they participated in the interventions or not. Our basic regression framework to estimate the ITT effect is:

$$Y_{i}=\beta_{0}+\beta_{1}T_{1}+\beta_{2}T_{2}+\varepsilon_{i}$$ (1)

*Y_i_* is the outcome variable for individual *i*, *T*_1_ and *T*_2_ are dummy variables which take the value 1 depending on which intervention the household in a village was assigned, 0 otherwise. The random error term is represented by *ε_i_*. The impacts of the interventions are given by *β*_1_ and *β*_2_. Standard errors are clustered at the village level to account for within village correlations. We conduct further analyses by extending Eq (1) to account for vectors of individual characteristics and other control variables, baseline and endline data, and treatment heterogeneity in terms of differences in the effects of the two interventions–*Treatment P* and *Treatment PC*. We assess any spillover effects by interviewing households who did not participate in the intervention. This assists in understanding whether and how the information regarding insect farming and consumption spread beyond the targeted households in the intervention [37].

### Ethical approval and consent to participate

Ethical approval is obtained from the Jaramogi Oginga Odinga University of Science and Technology, Ethics Review Office (Kenya) (approval number: **ERC 24/8/21-1S**), University of Natural Resources and Energy, School of Sciences, Committee for Human Research and Ethics (Ghana) (**“approved the protocol”**), and Makerere University, College of Humanities and Social Sciences, Research Ethics Committee (Uganda) (approval number: **MAKSSREC 12.21.524**). Written informed consent is obtained from all study participants.

### Trial status

Participant recruitment has been completed, and the intervention is ongoing at the time of submission. Follow-up data collection has not yet begun. The current protocol version is 2.0, dated 17 May 2023.

### Tentative study timeline

**Fig 4:**
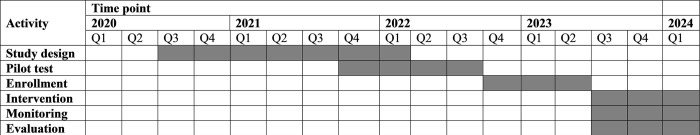
A tentative timeline for the intervention study.

A tentative timeline for the intervention study is presented in Fig 4.

## Discussion

Since the FAO (2013) publication [14], the notion of considering insects as food has received increasing attention. Edible insects are often associated with higher nutritional value and lower environmental impacts compared to many other animal-source proteins [10, 14, 18]. Insect farming is a means of producing “mini-livestock” without degrading the environment [54, 55]. This study protocol presents the first RCT study to evaluate the effectiveness of educational interventions in promoting insect farming and household consumption in Africa.

Previous observational and cross-sectional studies have suggested that the main constraints for insect farming in Africa is the lack of knowledge and skills [e.g. 21, 24]. While addressing these constraints through educational interventions is emphasized, no studies have so far investigated the causal impact of such interventions on insect farming using RCTs. In RCTs, individuals are randomly assigned to intervention and control arms to avoid biases observed in other study designs [56]. Specifically, RCTs ensure that any differences in intervention outcomes are entirely attributed to the intervention as randomization is done independent of observed and unobserved characteristics of individuals. Thus, the RCT enables us to provide a reliable evaluation of the impact of educational interventions on the primary and secondary outcomes. Given that our study is based on baseline and endline surveys accompanied by continuous intervention monitoring activities, it enables us to observe the changes resulting from the intervention and understand the mechanisms through which these changes occur [43]. This will allow us to generate findings involving the impact of the intervention, as well as the underlying mechanisms behind these impacts, including intervention elements, implementation, and monitoring.

Several studies conducted agricultural interventions in Africa using similar methodological approaches. For instance, Ogutu, Fongar [57] conducted an RCT in Kenya to evaluate the impact of agricultural and nutrition trainings on the adoption of pro-nutrition technologies such as bio-fortified crops. A related study by Blakstad, Mosha [49] investigated whether participation in agricultural, and nutrition and health counselling improves dietary diversity and quality through homestead food production in Tanzania. In Malawi, Kuchenbecker, Reinbott [58] assessed the effectiveness of nutrition education on child dietary diversity. Like our study, the three studies are based on cluster randomization, and they provide agricultural inputs to intervention participants although cash payment is required in Ogutu, Fongar [57]. Unlike ours and the first two studies, Kuchenbecker, Reinbott [58] used child anthropometric measures as outcome variables. The above studies discussed policy implications of their results, suggesting the importance of agricultural training and nutrition education in supporting nutrition improvement activities in Africa. The findings of our study are also useful for policy makers concerned with insect farming for sustainable food production and livelihoods in Africa. Our study is, thus, relevant and necessary in the context of insect farming in Africa, where insects are often appreciated as food and a source of livelihoods for many rural households.

Our focus on educational programs to promote the production and consumption of insects in Africa aligns with suggestions in the literature regarding strategies for promoting insects as a food source. As discussed in Maya, Sterling [59], education can play a key role in increasing the supply of sustainable foods through insect production, particularly in regions where insects are already considered a traditional food [5]. Additionally, this strategy can be applied in areas, notably Western countries, where insects are often associated with disgust. In such countries, education can help mitigate the perception of disgust and enhance willingness-to-eat insects by increasing interest in insects as food and fostering positive experiences regarding the idea of consuming insects [59, 60]. In addition to education, social influence can play a significant role in promoting insects as food, as indicated by review studies [61–63]. Social influence is also utilized as a strategy to increase the adoption of climate-smart agricultural practices in developing countries [35, 64]. Our study design enables us to investigate whether this factor plays a role in promoting insect farming and consumption. We ask baseline survey participants, who were not selected for the intervention, about their awareness and sources of information regarding insect farming and consumption. We also examine how this awareness influences their attitude and perception of insects as food, their willingness to adopt insect farming, and whether they have established an insect farm or consumed farmed insects.

The results of the study will be disseminated through various channels. Firstly, policy briefs will be prepared and presented to relevant stakeholders, including policymakers, government agencies, and the private sector. These briefs will provide a concise summary of the study’s findings and their implications for policy and decision-making. Secondly, we will organize public meetings in the study areas to present the results and engage in discussions regarding the importance of promoting insect farming for household consumption. Thirdly, we will prepare scientific articles for presentation at international conferences and publication in peer-reviewed journals. This will allow us to share the results with the academic community and contribute to the existing body of knowledge. Lastly, we will utilize social media platforms to share the results and reach diverse audiences with an interest in insects as food worldwide.

## Supporting information

S1 Checklist SPIRIT checklist for trials. (DOCX)

S1 File (DOC)

## References

[1] World Bank. Making development climate resilient: A world bank strategy for sub-saharan africa. 2009.

[2] Ringler C, Zhu T, Cai X, Koo J, Wang D. Climate change impacts on food security in sub-saharan africa. Insights from Comprehensive Climate Change Scenarios2010.

[3] OECD/FAO. Oecd-fao agricultural outlook 2016–2025. Paris: OECD Publishing; 2016.

[4] Parodi A, Leip A, De Boer IJM, Slegers PM, Ziegler F, Temme EHM, et al. The potential of future foods for sustainable and healthy diets. Nature Sustainability. 2018;1(12):782–9.

[5] Verner D, Roos N, Halloran A, Surabian G, Tebaldi E, Ashwill M, et al. Insect and hydroponic farming in africa: The new circular food economy: Washington, DC: World Bank; 2021.

[6] McKenzie FC, Williams J. Sustainable food production: Constraints, challenges and choices by 2050. Food Security. 2015;7(2):221–33.

[7] UN. Transforming our world: The 2030 agenda for sustainable development. United Nations: New York, NY, USA. 2015.

[8] Aiking H, Boer Jd. Protein and sustainability–the potential of insects. Journal of Insects as Food and Feed. 2019;5(1):3–7.

[9] Tanga CM, Egonyu JP, Beesigamukama D, Niassy S, Emily K, Magara HJO, et al. Edible insect farming as an emerging and profitable enterprise in east africa. Current Opinion in Insect Science. 2021;48:64–71. doi: 10.1016/j.cois.2021.09.007 34649017

[10] Huis A, Rumpold B, Maya C, Roos N. Nutritional qualities and enhancement of edible insects. Annual Review of Nutrition. 2021;41(1):551–76. doi: 10.1146/annurev-nutr-041520-010856 34186013

[11] Halloran A, Roos N, Eilenberg J, Cerutti A, Bruun S. Life cycle assessment of edible insects for food protein: A review. Agronomy for Sustainable Development. 2016;36(4):57. doi: 10.1007/s13593-016-0392-8 32010238 PMC6961468

[12] Alexander P, Brown C, Arneth A, Dias C, Finnigan J, Moran D, et al. Could consumption of insects, cultured meat or imitation meat reduce global agricultural land use? Global Food Security. 2017;15:22–32.

[13] Lipper L, Thornton P, Campbell BM, Baedeker T, Braimoh A, Bwalya M, et al. Climate-smart agriculture for food security. Nature Climate Change. 2014;4(12):1068–72.

[14] FAO. Edible insects: Future prospects for food and feed security: Food and Agriculture Organization of the United Nations; 2013.

[15] Costa-Neto EM, Dunkel FV. Chapter 2—insects as food: History, culture, and modern use around the world. In: Dossey AT, Morales-Ramos JA, Rojas MG, editors. Insects as sustainable food ingredients. San Diego: Academic Press; 2016. p. 29–60.

[16] YEN AL. Edible insects: Traditional knowledge or western phobia? Entomological Research. 2009;39(5):289–98.

[17] Gahukar RT. Edible insects collected from forests for family livelihood and wellness of rural communities: A review. Global Food Security. 2020;25:100348.

[18] Huis A, Oonincx DGAB. The environmental sustainability of insects as food and feed. A review. Agronomy for Sustainable Development. 2017;37(5):43.

[19] Niyonsaba HH, Höhler J, Kooistra J, Fels-Klerx HJVd, Meuwissen MPM. Profitability of insect farms. Journal of Insects as Food and Feed. 2021;7(5):923–34.

[20] Wilderspin DE, Halloran A. The effects of regulation, legislation and policy on consumption of edible insects in the global south. Edible insects in sustainable food systems: Springer; 2018. p. 443–55.

[21] Nischalke S, Wagler I, Tanga C, Allan D, Phankaew C, Ratompoarison C, et al. How to turn collectors of edible insects into mini-livestock farmers: Multidimensional sustainability challenges to a thriving industry. Global Food Security. 2020;26:100376.

[22] Kinyuru J, Ndung’u N. Edible insects regulatory national standards in kenya: An incentive or a deterrent? Journal of Agriculture, Science and Technology. 2022;21(4):1–3.

[23] Berggren Å, Jansson A, Low M. Using current systems to inform rearing facility design in the insect-as-food industry. Journal of Insects as Food and Feed. 2018;4(3):167–70.

[24] Durst PB, Hanboonsong Y. Small-scale production of edible insects for enhanced food security and rural livelihoods: Experience from thailand and lao people’s democratic republic. Journal of Insects as Food and Feed. 2015;1(1):25–31.

[25] Halloran A, Roos N, Hanboonsong Y. Cricket farming as a livelihood strategy in thailand. The Geographical Journal. 2017;183(1):112–24.

[26] Ayieko MA, Ogola HJ, Ayieko IA. Introducing rearing crickets (gryllids) at household levels: Adoption, processing and nutritional values. Journal of Insects as Food and Feed. 2016;2(3):203–11.

[27] Belluco S, Losasso C, Maggioletti M, Alonzi CC, Paoletti MG, Ricci A. Edible insects in a food safety and nutritional perspective: A critical review. Comprehensive Reviews in Food Science and Food Safety. 2013;12(3):296–313.

[28] Halloran A, Ayieko M, Oloo J, Konyole SO, Alemu MH, Roos N. What determines farmers’ awareness and interest in adopting cricket farming? A pilot study from kenya. International Journal of Tropical Insect Science. 2021;41(3):2149–64.

[29] Raheem D, Carrascosa C, Oluwole OB, Nieuwland M, Saraiva A, Millán R, et al. Traditional consumption of and rearing edible insects in africa, asia and europe. Critical Reviews in Food Science and Nutrition. 2019;59(14):2169–88. doi: 10.1080/10408398.2018.1440191 29446643

[30] Malinga GM, Valtonen A, Hiltunen M, Lehtovaara VJ, Nyeko P, Roininen H. Performance of the african edible bush-cricket, ruspolia differens, on single and mixed diets containing inflorescences of their host plant species. Entomologia Experimentalis et Applicata. 2020;168(6–7):448–59.

[31] Adams F, Aidoo R, Mensah JO, Mensah A, Amankwah K, Kyei BK, et al. Commercialisation of african palm weevil larvae for employment creation and nutritional security in rural ghana: A financial feasibility approach. Journal of Insects as Food and Feed. 2021;7(6):1051–60.

[32] Kanter R, Walls HL, Tak M, Roberts F, Waage J. A conceptual framework for understanding the impacts of agriculture and food system policies on nutrition and health. Food Security. 2015;7(4):767–77.

[33] Webb P. Impact pathways from agricultural research to improved nutrition and health: Literature analysis and research priorities. Rome: Food and Agriculture Organization and Geneva: World Health Organization. 2013.

[34] Commander T, Anankware JP, Royal OO, Obeng-Ofori D. Econometrics of domestication of the african palm weevil (rhynchophorus phoenicis f.) production as small-scale business in ghana. Edible insects: IntechOpen; 2019.

[35] Shikuku KM. Information exchange links, knowledge exposure, and adoption of agricultural technologies in northern uganda. World Development. 2019;115:94–106.

[36] Raudenbush et al. Optimal design software for multi-level and longitudinal research (version 3.01) [software]. 3.01 ed2011.

[37] de Brauw A, Eozenou P, Gilligan DO, Hotz C, Kumar N, Meenakshi JV. Biofortification, crop adoption and health information: Impact pathways in mozambique and uganda. American Journal of Agricultural Economics. 2018;100(3):906–30. doi: 10.1093/ajae/aay005 32139914 PMC7053385

[38] Shikuku KM, Okello JJ, Wambugu S, Sindi K, Low JW, McEwan M. Nutrition and food security impacts of quality seeds of biofortified orange-fleshed sweetpotato: Quasi-experimental evidence from tanzania. World Development. 2019;124:104646. doi: 10.1016/j.worlddev.2019.104646 31798205 PMC6876675

[39] Takahashi K, Muraoka R, Otsuka K. Technology adoption, impact, and extension in developing countries’ agriculture: A review of the recent literature. Agricultural Economics. 2020;51(1):31–45.

[40] Chavas J-P, Nauges C. Uncertainty, learning, and technology adoption in agriculture. Applied Economic Perspectives and Policy. 2020;42(1):42–53.

[41] StataCorp LJCS, TX: StataCorp LP. Stata statistical software: Release 18. 2023.

[42] Michie S, van Stralen MM, West R. The behaviour change wheel: A new method for characterising and designing behaviour change interventions. Implementation Science. 2011;6(1):42. doi: 10.1186/1748-5908-6-42 21513547 PMC3096582

[43] Wendt AS, Sparling TM, Waid JL, Mueller AA, Gabrysch S. Food and agricultural approaches to reducing malnutrition (faarm): Protocol for a cluster-randomised controlled trial to evaluate the impact of a homestead food production programme on undernutrition in rural bangladesh. BMJ Open. 2019;9(7):e031037. doi: 10.1136/bmjopen-2019-031037 31278109 PMC6615849

[44] Baiano A. Edible insects: An overview on nutritional characteristics, safety, farming, production technologies, regulatory framework, and socio-economic and ethical implications. Trends in Food Science & Technology. 2020;100:35–50.

[45] Kondylis F, Mueller V, Zhu S. Measuring agricultural knowledge and adoption. Agricultural Economics. 2015;46(3):449–62.

[46] Shikuku KM, Pieters J, Bulte E, Läderach P. Incentives and the diffusion of agricultural knowledge: Experimental evidence from northern uganda. American Journal of Agricultural Economics. 2019;101(4):1164–80.

[47] Caeiro RM, Vicente PC. Knowledge of vitamin a deficiency and crop adoption: Evidence from a field experiment in mozambique. Agricultural Economics. 2020;51(2):175–90.

[48] Bandoh DA, Kenu E. Dietary diversity and nutritional adequacy of under-fives in a fishing community in the central region of ghana. BMC Nutrition. 2017;3(1):2.

[49] Blakstad MM, Mosha D, Bellows AL, Canavan CR, Chen JT, Mlalama K, et al. Home gardening improves dietary diversity, a cluster-randomized controlled trial among tanzanian women. Maternal & Child Nutrition. 2021;17(2):e13096. doi: 10.1111/mcn.13096 33241924 PMC7988851

[50] Cafiero C, Viviani S, Nord M. Food security measurement in a global context: The food insecurity experience scale. Measurement. 2018;116:146–52.

[51] Dohmen T, Falk A, Huffman D, Sunde U, Schupp J, Wagner GG. Individual risk attitudes: Measurement, determinants, and behavioral consequences. Journal of the European Economic Association. 2011;9(3):522–50.

[52] Asravor RK. Farmers’ risk preference and the adoption of risk management strategies in northern ghana. Journal of Environmental Planning and Management. 2019;62(5):881–900.

[53] Arslan RC, Brümmer M, Dohmen T, Drewelies J, Hertwig R, Wagner GG. How people know their risk preference. Scientific Reports. 2020;10(1):15365. doi: 10.1038/s41598-020-72077-5 32958788 PMC7505965

[54] Av Huis. Potential of insects as food and feed in assuring food security. Annual Review of Entomology. 2013;58(1):563–83. 10.1146/annurev-ento-120811-153704 23020616

[55] Tabassum A, Abbasi T, Abbasi SA. Reducing the global environmental impact of livestock production: The minilivestock option. Journal of Cleaner Production. 2016;112:1754–66.

[56] Duflo E, Glennerster R, Kremer M. Chapter 61 using randomization in development economics research: A toolkit. In: Schultz TP, Strauss JA, editors. Handbook of development economics. 4: Elsevier; 2007. p. 3895–962.

[57] Ogutu SO, Fongar A, Gödecke T, Jäckering L, Mwololo H, Njuguna M, et al. How to make farming and agricultural extension more nutrition-sensitive: Evidence from a randomised controlled trial in kenya. European Review of Agricultural Economics. 2018;47(1):95–118.

[58] Kuchenbecker J, Reinbott A, Mtimuni B, Krawinkel MB, Jordan I. Nutrition education improves dietary diversity of children 6–23 months at community-level: Results from a cluster randomized controlled trial in malawi. PLOS ONE. 2017;12(4):e0175216. doi: 10.1371/journal.pone.0175216 28426678 PMC5398527

[59] Maya C, Sterling K, Rukov JL, Roos N. Perception of edible insects and insect-based foods among children in denmark: Educational and tasting interventions in online and in-person classrooms. Journal of Insects as Food and Feed.0(0):1–14.

[60] Looy H, Dunkel FV, Wood JR. How then shall we eat? Insect-eating attitudes and sustainable foodways. Agriculture and Human Values. 2014;31(1):131–41.

[61] Wendin KME, Nyberg ME. Factors influencing consumer perception and acceptability of insect-based foods. Current Opinion in Food Science. 2021;40:67–71.

[62] Kröger T, Dupont J, Büsing L, Fiebelkorn F. Acceptance of insect-based food products in western societies: A systematic review. Frontiers in Nutrition. 2022;8. doi: 10.3389/fnut.2021.759885 35265649 PMC8901202

[63] Anusha Siddiqui S, Bahmid NA, Mahmud CMM, Boukid F, Lamri M, Gagaoua M. Consumer acceptability of plant-, seaweed-, and insect-based foods as alternatives to meat: A critical compilation of a decade of research. Critical Reviews in Food Science and Nutrition. 2022:1–22. doi: 10.1080/10408398.2022.2036096 35144515

[64] BenYishay A, Mobarak AM. Social learning and incentives for experimentation and communication. The Review of Economic Studies. 2018;86(3):976–1009.

