# Supplementary material for: Promoting insect farming and household consumption through agricultural training and nutrition education in Africa: A study protocol for a multisite cluster-randomized controlled trial
Source: PLoS One. 2023 Jul 19;18(7):e0288870. doi: 10.1371/journal.pone.0288870 (PMC10355422; doi:10.1371/journal.pone.0288870)
Supplement: S1 File — (DOC) [file pone.0288870.s002.doc]

**Title of study**

HEALTHYNSECT – Insect farming for health and livelihoods in Africa

**Principal investigator**Principal investigator’s name and address in Ghana, Kenya and Uganda

Principal investigator’s telephone number and email address in Ghana, Kenya and Uganda

**Information Sheet**

[Institution] is conducting research on promoting insect farming for household consumption in [Country]. This involves training farmers on insect farming techniques, as well as educating them on cooking and consuming insects. We are providing you with this information because we would like to invite you to participate in our research project. However, please note that your participation is voluntary, and you are free to choose whether or not to participate in all or part of the research. There will be no negative impact if you choose not to participate. We want to ensure that you have all the necessary information before making a decision. If you have any questions or need further clarification, please feel free to ask the researcher.

**Why the project is important?**

Insects are essential for human nutrition as they provide protein and other vital nutrients. They can be produced using cost-effective, locally available materials like organic waste and require less land and water compared to livestock. This enables year-round insect production, even during drought periods. Insect farming has the potential to enhance household livelihoods by generating additional income and creating employment opportunities for household members. The objective of this study is to promote insect farming and consumption in [Country]. To achieve this, we will provide training to farmers on effective insect farming techniques, as well as educate them on the methods of cooking and incorporating insects into their households. The study aims to assess the extent to which the training facilitates sustained insect farming and promotes insect consumption.

**Who can participate?**

You are being invited to take part in this research project because you participated in the baseline survey and it is on that basis you are contacted for further interventions on insect farming and consumption.

**What is involved in the project?**

If you agree to participate in the research, the following will happen

1. You will be invited to attend a training session at which some training is given on insect farming and consumption.
2. You will be assigned to group A, B or C. All households in your village that are participating in this study will be assigned to the same group. We have assigned your village to one of these groups by random selection, like flipping a coin.
3. If you are in group A, you will participate in training on farming insects. You will learn how to construct insect farming house; how to give feed and water for the insects; how to protect them from pest and disease; how to care eggs and young insects, how to maintain hygiene during insect farming; how to harvest the insects; how to store and transport them; and how to use the harvested insects. After the training, you will be offered materials for free to start the insect farming. We will visit you in about a week time after the training to check how things are going related to your insect farming. Thereafter, you will be visited at least once a month.
4. If you are in group B, you will participate in an additional session on top of the above training. You will receive information on why insects are important for human consumption and participate in pre-processes, cooking and tasting of the insect food. During food demo, you will participate or observe practical demonstrations on insect cooking and consumption under leadership of a trained research team leader. We will visit you in about a week time after this session to check how things are going related to your insect farming and consumption in the household. Thereafter, you will be visited at least once a month.
5. If you are in group C, nothing will change and you will not participate in trainings related to insect farming and consumption.
6. We will hold a meeting to explain the results of the study in the villages, and these will be announced. Following the meetings, we will publish the results so that other interested people may learn from the research.

If changes are made in the study or new information becomes available, you will be informed.

**What am I consenting to?**

If you are assigned to group A or B, you will consent to:

1. Work with the research team in answering various questions through interviews
2. Participate in insect farming
3. Participate in insect food cooking and consumption

**How long will I be in the project?**

The study will last for about 6 months.

**What are the risk of the study?**

There are minimal to no anticipated physical risks or side effects associated with this study. In the event of any allergic reactions that may occur when consuming a new food, appropriate monitoring and treatment will be provided at the nearest health facility. While there is a small possibility of inadvertently sharing personal or confidential information, we are committed to safeguarding your privacy. Data will only be accessed by authorized research staff solely for research purposes. If you feel uncomfortable discussing certain topics or sharing personal information, you have the right to decline to answer any or all questions and can terminate your involvement in the study at any time. We understand that discussing sensitive topics may make you uncomfortable, and we assure you that you will not be victimized or denied any other services provided to other participants in the program if you choose to decline participating in food preparation, cooking, or tasting. Your information will be treated with utmost confidentiality, and we will make every effort to ensure your privacy is protected.

**What are the benefits of the study?**

Insect farming offers various benefits that can positively impact you and your household. By participating in this study, you will receive free materials to initiate your insect farming venture. The insects produced can be utilized as a food source within your household, ensuring a continuous supply of food throughout the year, even during drought conditions. Furthermore, you have the opportunity to generate income by selling the insects to others. Insect farming also provides employment opportunities for you and members of your household, contributing to improved livelihoods. Your participation in this study is invaluable as it contributes to our understanding and promotion of insect farming for household consumption in [Country]. Through your involvement, we can gather valuable insights and information to enhance the practices and benefits of insect farming in local communities

**How will we keep confidentiality?**

The research being done in the village may draw attention and if you participate, other people in the village may ask you questions. Your responses to this study will be anonymous. Every effort will be made by the researcher to preserve your confidentiality including the following:

- We will not be sharing information about you or your household to anyone outside of the research team.
- The information that we collect from this research project will be kept private.
- Any information about you will have a number on it instead of your name. Only the researchers will know what your number is and we will lock that information up with a lock and key. It will not be shared with or given to anyone outside of our project.

**Who can I contact?**

If you have any questions at any time about this study, you can ask anyone from our team now or later. If you have questions later, you may contact [principal investigator’s telephone number and email].

For questions about your rights as a research participant, or to discuss problems, concerns or suggestions related to the research, or to obtain information or offer input about the research, contact the [the responsible research institution’s telephone number in Ghana, Kenya and Uganda].

**Voluntary participation**

Your participation in this study is voluntary. It is up to you to decide whether or not to take part in this study. If you decide to take part in this study, you will be asked to sign a consent form. After you sign the consent form, you are still free to withdraw at any time and without giving a reason. Withdrawing from this study will not affect the relationship you have, if any, with the researcher. If you do decide to withdraw, we ask that you contact study staff to let them know you want to withdraw. If you choose to stop taking part, you will continue to get all of the normal services that you usually get in your community.

**Statement of consent**

I have read the above information or it has been read to me**,** and I understand the provided information and have had the opportunity to ask questions. I understand that my participation is voluntary and that I am free to withdraw at any time, without giving a reason and without cost. I understand that I will be given a copy of this consent form. I voluntarily agree to take part in this study.

Participant's signature ______________________________ Date __________

Investigator's signature _____________________________ Date __________
